# Incidental Ectopic Gallbladder Mimicking an Epigastric Cyst

**DOI:** 10.7759/cureus.91742

**Published:** 2025-09-06

**Authors:** Teferi G Gebreslassie, Dori W Abraha

**Affiliations:** 1 Diagnostic Radiology, Manhal Specialty Hospital, Hargeisa, SOM; 2 Diagnostic Radiology, Partners in Health, Burera, RWA

**Keywords:** computed tomography, ectopic gallbladder, epigastric cyst, incidental gallbladder anomalies, ultrasound

## Abstract

Ectopic gallbladder is a rare congenital anomaly with important clinical and surgical implications. Although it is normally situated on the undersurface of the right hepatic lobe, it can occasionally be found in unusual locations such as intrahepatic, left-sided, within the lesser omentum, retroperitoneal, falciform ligament, or anterior abdominal wall. We describe the case of a 60-year-old male from Somaliland who presented with rectal bleeding attributed to hemorrhoids, during which an incidental ectopic gallbladder was identified. Initial ultrasound suggested an epigastric cyst between the left hepatic lobe and the lesser curvature of the stomach, with no associated hepatobiliary pathology. Subsequent contrast-enhanced computed tomography confirmed the presence of an ectopic gallbladder located in the left subhepatic space. Accurate recognition of this rare anomaly on imaging is essential to prevent misdiagnosis and to ensure appropriate surgical planning. This case underscores the pivotal role of radiologists in identifying gallbladder anatomical variations with potential clinical and management significance.

## Introduction

The gallbladder is usually located in the gallbladder fossa on the visceral surface of the right hepatic lobe. Ectopic gallbladder is a rare congenital anomaly, with a reported incidence of less than 0.1% [1]. Common ectopic locations include intrahepatic, left-sided, within falciform ligament, within lesser omentum, retroperitoneum and anterior abdominal wall [2]. We present an incidentally discovered ectopic gallbladder in a 60-year-old male patient.

## Case presentation

A 60-year-old male from Somaliland presented with a recurrent onset of lower gastrointestinal bleeding. On physical examination, there were swollen bluish anal lumps with tenderness and no masses on digital rectal examination.

**Figure 1:**
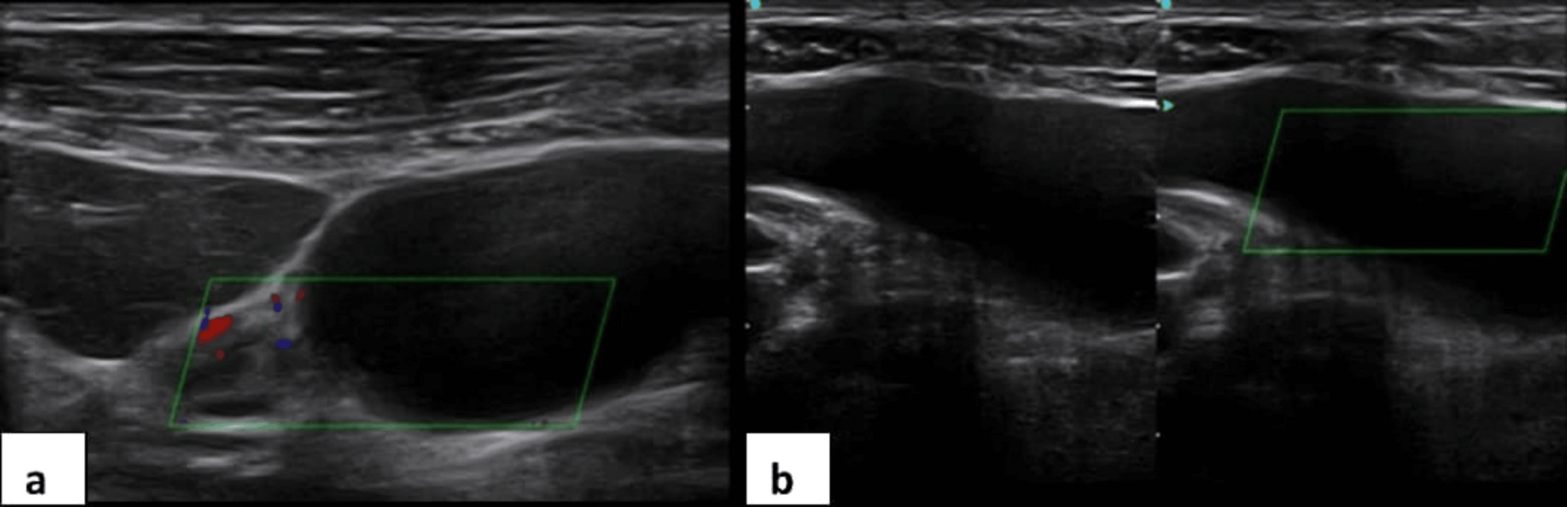
Ultrasound images showing an epigastric cyst later identified as an ectopic gallbladder Ultrasound images showing an epigastric cyst with no color flow located medial to the hepatic lobe, which was later identified as ectopic gallbladder, showing part of the body, neck, and cystic duct (a) and body and fundus (b)

On initial ultrasound, a cystic lesion was identified in the epigastric region with no color flow, located between the left hepatic lobe and the lesser curvature of the stomach (Figure 1). This finding led to a preliminary impression of an epigastric cyst, partly because the epigastric region is typically the first scanned area, with a differential of diagnosis that included a duplication cyst or pancreatic pseudocyst. However, further ultrasound evaluation of the right upper quadrant revealed an empty gallbladder fossa. The liver, hepatobiliary tree, pancreas, and the rest of the abdominal viscera were unremarkable.

**Figure 2:**
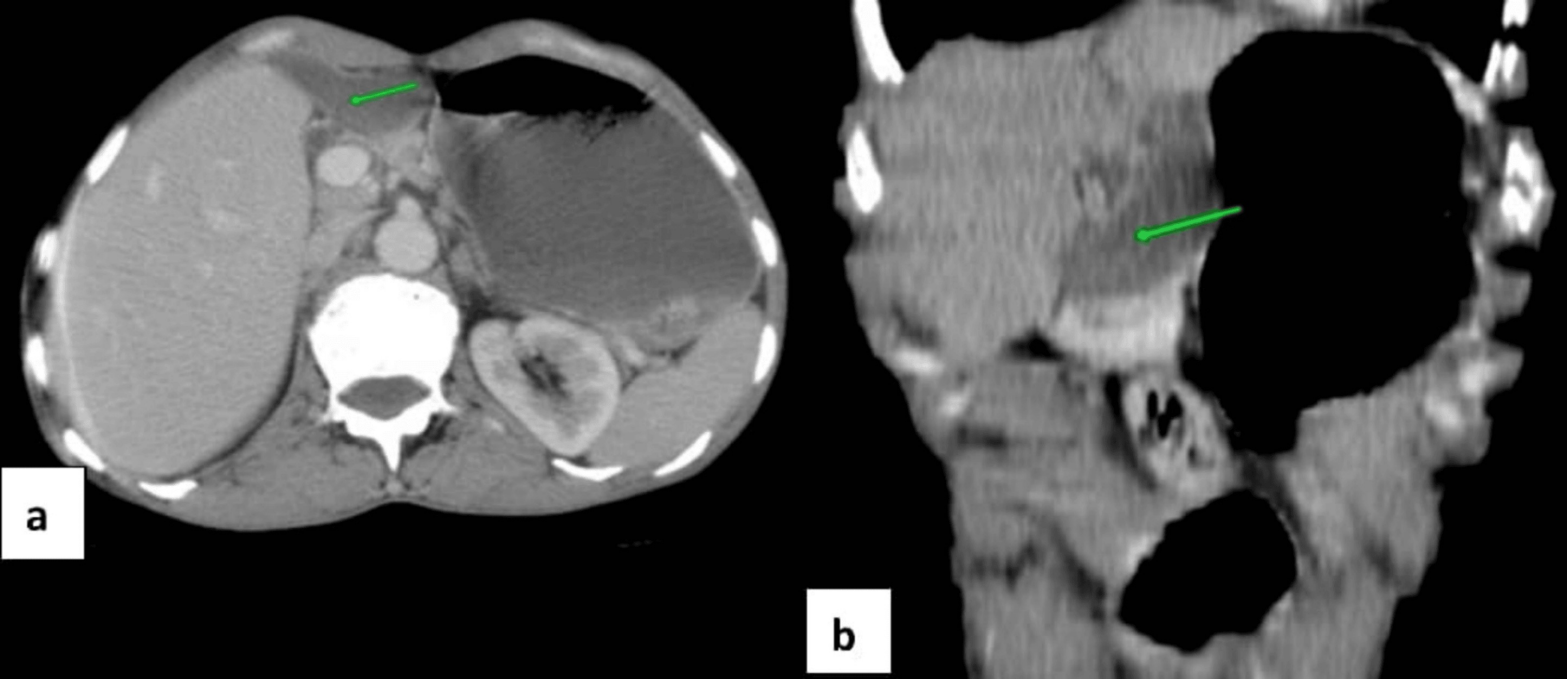
CT abdomen images demonstrating aberrant gallbladder position in the left subhepatic space Contrast-enhanced axial (a) and coronal (b) CT scans confirming the anomalous location of the gallbladder in the left subhepatic space (arrow indicates gallbladder)

Subsequent contrast-enhanced computed tomography (CT) confirmed an ectopic gallbladder, situated in the left subhepatic space (Figure 2). Imaging demonstrated an unremarkable gallbladder, with absence of gallstones, normal wall thickness, no pericholecystic fluid, and no evidence of associated hepatobiliary pathology.

Lower gastrointestinal bleeding was attributed to external hemorrhoids, and ectopic gallbladder was considered an incidental finding.

## Discussion

An ectopic gallbladder is very uncommon, with less than 0.1% of cases documented in the literature [1]. This condition arises from abnormal migration of the gallbladder bud during embryogenesis [2]. Congenital malformations of the gallbladder can be classified based on their size, location, number, and shape, with the most prevalent congenital anomaly being a variation in the location [3].

Ultrasonography can only identify abnormal positions of the gallbladder in approximately 16.3% of cases [4], and the vast majority of cases (>80%) are found during surgical procedures [3]. Although frequently asymptomatic, an abnormal gallbladder location can complicate the diagnosis of hepatobiliary diseases and lead to complications such as cholelithiasis, torsion, or errors in imaging diagnostics. Therefore, it is crucial to note that surgical interventions in the hepatobiliary or upper abdominal areas present an increased risk of inadvertent injuries if the anomaly remains unrecognized.

This case emphasizes the need for radiologists to perform comprehensive abdominal assessments and to recognize atypical presentations, even when imaging is conducted for indications unrelated to the hepatobiliary system.

## Conclusions

Ectopic gallbladder is a rare congenital anomaly that may present as an incidental finding during imaging performed for unrelated conditions. Although often asymptomatic, its atypical location can mimic other abdominal pathologies and complicate both radiologic interpretation and surgical management. This case emphasizes the importance of careful and systematic imaging review, particularly when evaluating cystic lesions in unusual locations. Awareness of ectopic gallbladder and other anatomic variants is essential for anticipating complications, preventing intraoperative injury, and guiding safe management toward optimal patient outcomes.
